# Biphasic Calcium Phosphate Bone Graft With a Unique Surface Topography: A Single-Center Ambispective Study for Degenerative Disease of the Lumbar Spine

**DOI:** 10.7759/cureus.58218

**Published:** 2024-04-13

**Authors:** Pierce Nunley, Milo Sanda, Henry Googe, David Cavanaugh, Katherine Sage, James Ryaby, Marcus B Stone

**Affiliations:** 1 Spine Surgery, Spine Institute of Louisiana, Shreveport, USA; 2 Spine, Spine Institute of Louisiana, Shreveport, USA; 3 Neurosurgery, Spine Institute of Louisiana, Shreveport, USA; 4 Orthopedic Surgery, Kuros Biosciences USA, Inc., Atlanta, USA; 5 Spine, Kuros Biosciences USA, Inc., Atlanta, USA

**Keywords:** low back pain, bone graft, interbody fusion, degenerative, lumbar

## Abstract

Study design: This study is an ambispective evaluation and analysis of a single-center cohort.

Objective: This study aimed to evaluate the performance of a novel biphasic calcium phosphate (BCP) bone graft with submicron-sized needle-shaped surface topography (BCP<µm) in interbody arthrodesis of the lumbar spine.

Methods: This study was a single-center ambispective assessment of adult patients receiving BCP<µm as part of their lumbar interbody fusion surgery. The primary outcome was a fusion status on computed tomography (CT) 12 months postoperative. The secondary outcomes included postoperative changes in the visual analog scale (VAS), Oswestry Disability Index (ODI), Short Form 12 (SF-12), and length of stay (LOS).

Results: Sixty-three patients with one- to three-level anterior (48, 76%) and lateral (15, 24%) interbody fusions with posterior instrumentation were analyzed. Thirty-one participants (49%) had three or more comorbidities, including heart disease (43 participants, 68%), obesity (31 participants, 49%), and previous lumbar surgery (23 participants, 37%). The mean ODI decreased by 24. The mean SF-12 physical health and SF-12 mental health improved by a mean of 11.5 and 6.3, respectively. The mean VAS for the left leg, right leg, and back improved by a mean of 25.75, 22.07, and 37.87, respectively. Of 101 levels, 91 (90%) demonstrated complete bridging trabecular bone fusion with no evidence of supplemental fixation failure.

Conclusion: The data of BCP<µm in interbody fusions for degenerative disease of the lumbar spine provides evidence of fusion in a complicated cohort of patients.

## Introduction

Iliac crest bone graft (ICBG) is considered the gold standard bone graft, but with limited availability and associated comorbidities, spine surgeons often utilize alternative bone grafts. One challenge for surgeons comes in identifying the most scientifically advanced bone graft alternative to avoid added operating time and the known complications inherent in harvesting autograft [1]. 

Bone graft substitutes can be classified into two main categories: bone substitutes derived from biological products and synthetic bone substitutes. Biologic products include demineralized bone matrix (DBM), platelet-rich plasma (PRP), hydroxyapatite (HA), coralline HA, and cellular bone allografts (CBAs) [2]. Synthetic bone graft substitutes include calcium sulfate, calcium phosphate (CaP) cement, β-tri-calcium phosphate ceramics (β-TCP), biphasic calcium phosphate (BCP), bioactive glasses, and polymer-based bone substitutes [3]. 

Allogenic bone in the form of DBMs and CBAs has traditionally been used as an alternative to autograft because this circumvents the morbidity of autograft harvest. There is, however, a risk of an immunogenic response and disease transmission [4]. Procedures for minimizing this risk can alter the biological and mechanical properties by freezing or irradiation, which decreases the osteoinductive capabilities of the original graft tissue [5]. An additional disadvantage of allograft is the variability in graft quality [6]. The concerns over the use of either autograft or allograft have led to the development of numerous bone graft substitutes.

CaPs are synthetic bone graft substitutes. The synthetic CaP bone graft closely resembles human cancellous bone and has an exceptional safety profile, which has proven to be a cost-effective alternative to autograft [7-9]. Historically, CaP bone grafts were used off-label for interbody fusions, but recently, some CaPs have gained FDA clearance in this indication. MagnetOs Easypack Putty and MagnetOs Flex Matrix (Kuros Biosciences, Bilthoven, NL), Catalyst (OssDsign, Uppsala, Sweden), Fibergraft™ (Prosidyan, New Providence, NJ, USA), and Attrax (NuVasive, San Diego, CA, USA) have recently received FDA approved for utilization in interbody fusion devices [10,11]. However, prior to this paper, there are limited published data on the performance of these CaPs in human clinical trials.

Most conventional CaPs are osteoconductive, but a subset of CaPs have demonstrated osteoinductive properties, triggering de novo bone formation in sites distant from the host bone [12]. The reason for the improved osteoinductive capabilities has been linked to an advanced submicron surface topography. These CaPs with submicron surface topography have been shown to accelerate bone formation and are equivalent to autograft in clinically relevant animal models [13-15]. In various preclinical studies, CaPs with submicron topography have been shown to be more effective than CaPs without this surface topography [16,17].

The body’s natural response to tissue injury caused by spinal surgery is the upregulation of pro-inflammatory macrophages (“classically activated M1 macrophages”), which are the first responders of the immune system. If that inflammatory response becomes chronic, there is a risk of fibrosis, non-union, and the development of pseudarthrosis in spine fusion defects. The unique needle-shaped submicron surface of this novel BCP (BCP<µm) polarizes naïve monocytes to the pro-healing and anti-inflammatory M2 phenotype of macrophage, which leads to the upregulation of mesenchymal stromal cells and the formation of bone rather than the scar tissue [18].

In preclinical studies, this BCP<µm has been shown to promote bone formation, even in soft tissues, without the need for added cells or growth factors [18]. This novel BCP<µm bone graft is designed to mimic the porous, trabecular structure of the cancellous bone, and bone formation takes place throughout the BCP<µm bone graft, leading to a uniform, solid, and stable fusion, which has been found in clinically relevant animal studies [13,14,18]. In addition, recent level one clinical data of a prospective, randomized controlled trial show a fusion rate of 78% of this BCP<µm bone graft in an open instrumented posterolateral fusion model [19]. 

The ability of this BCP<µm with both osteoconductive and osteoinductive capabilities through their unique submicron needle-shaped surface topography has been demonstrated to be effective in posterolateral fusions [20] and makes it a viable alternative to autograft harvest. The objective of this ambispective study with prospective radiographic and patient-reported outcomes is to assess the performance of a novel biphasic CaP bone graft with submicron-sized needle-shaped surface topography (BCP<µm) (MagnetOs Putty, Kuros Biosciences, Atlanta, GA) in an interbody fusion model for degenerative disease of the lumbar spine.

## Materials and methods

Study design and patients

This ambispective evaluation included a retrospective review of medical records for 95 patients at a single center, the Spine Institute of Louisiana, Shreveport, LA. Patients who underwent lumbar arthrodesis for degenerative disease utilizing BCP<µm were invited to participate. Beyond the retrospective chart review, study participation included a one-time prospective evaluation with a computed tomography (CT) scan and X-rays at approximately 12 months postoperative. The method of arthrodesis was at the discretion of the treating surgeon and included both anterior and lateral lumbar interbody fusion with minimally invasive posterior percutaneous pedicle screw instrumentation. No posterior bone grafting was performed. All surgeries were performed by two fellowship-trained, board-certified orthopedic spine surgeons. The individual surgical indications included retrospondylolisthesis, spondylolisthesis, lumbar scoliosis, spinal stenosis, and spondylosis with degenerative disc disease. All procedures were performed between August 2019 and December 2020.

The inclusion criteria for the study included clinical or radiographic evidence of degenerative disc disease of the lumbar spine, treated with BCP<µm, and at least 18 years of age. Potential participants were also willing and able to return to the clinic for a CT scan and X-rays and complete patient-reported outcome questionnaires. Potential participants were excluded from the study if they were currently imprisoned, experiencing major mental illness (psychosis, schizophrenia, and major affective disorder), contraindicated to receive X-rays or CT scan, or had any previous lumbar fusion or arthroplasty surgery at the index levels.

Methods

After IRB approval, the retrospectively identified patients were contacted by telephone. The study was explained in brief. If patients were amenable to learn more about the study, they were asked to come to the clinic for a prospective visit. After reporting to the clinic, the study was further explained and informed consent was obtained from those willing to participate. The patients then underwent a non-contrast CT scan and radiographs. The CT was performed using a Hitachi Supria True64 CT scanner (Hitachi, Ltd., Japan) with a 3 mm slice thickness as is routine clinical practice to assess fusion. X-rays of the lumbar spine included anteroposterior and lateral views in the flexion and extension positions and were performed using a Carestream Z-Rad-DRX-1 (Carestream, USA).

From there, the participants completed a visual analog scale (VAS) pain score for the left leg, right leg, and back, an Oswestry Disability Index (ODI) survey, and a Short Form 12 (SF-12) questionnaire. These prospectively collected patient-centered outcome measures were used to compare with the same measures that were collected from them preoperatively as part of their normal clinical care. In addition, subject demographics, medical history, social history, and post-surgical care data were collected by research staff to determine factors that may affect the performance of BCP<µm. Specifically, comorbid conditions include obesity, tobacco smoking, diabetes, previous spine surgery, heart disease, thyroid disease, respiratory disease, and cancer. The body mass index (BMI) was categorized according to the National Institutes of Health (NIH) standards as underweight (<18.5), normal (18.5-24.9), overweight (≥25 and <30), or obese (≥30).

The primary outcome was determined by the fusion status on CT and four-view radiographs at 12 or more months postoperative, interpreted by a blinded, independent, board-certified neurosurgeon. Patient participants were deemed fused when there was evidence of bridging trabecular bone on the CT scan and less than three degrees of angular motion when comparing flexion and extension lateral radiographs.

Secondary outcomes included baseline to postoperative changes in ODI, VAS for pain, and SF-12 physical and mental scores. Changes in neurologic status from baseline to postoperative visits, adverse events, complications considered to be related to BCP<µm within 12 months post-surgery, and the number of patients that underwent revision/reoperation within 12 months post-surgery were also collected.

Statistical analysis

Statistical analysis was performed by utilizing descriptive statistics for continuous variables consisting of the mean, median, standard deviation, and minimum and maximum values. For categorical variables, the number and percentage of each category were analyzed.

## Results

**Figure 1:**
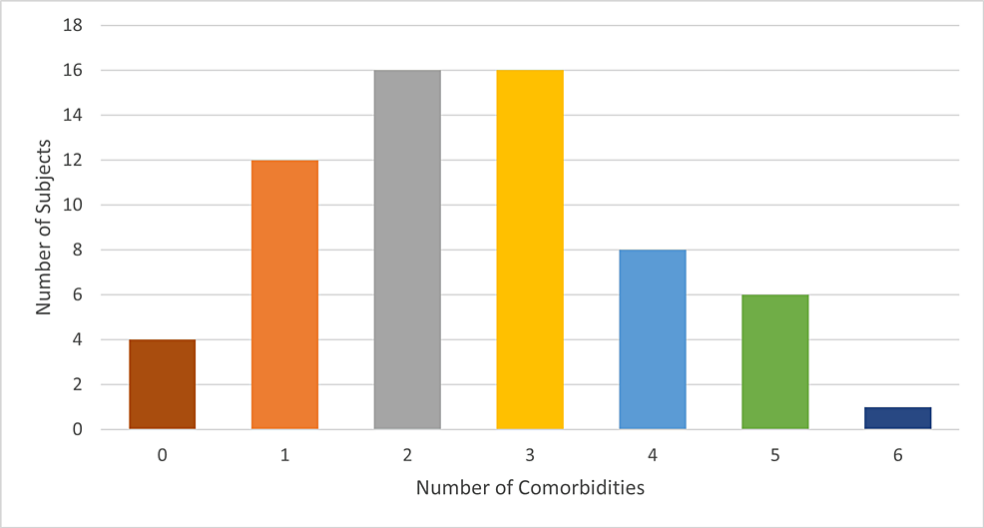
Number of comorbidities per patient. n = 63

**Figure 2:**
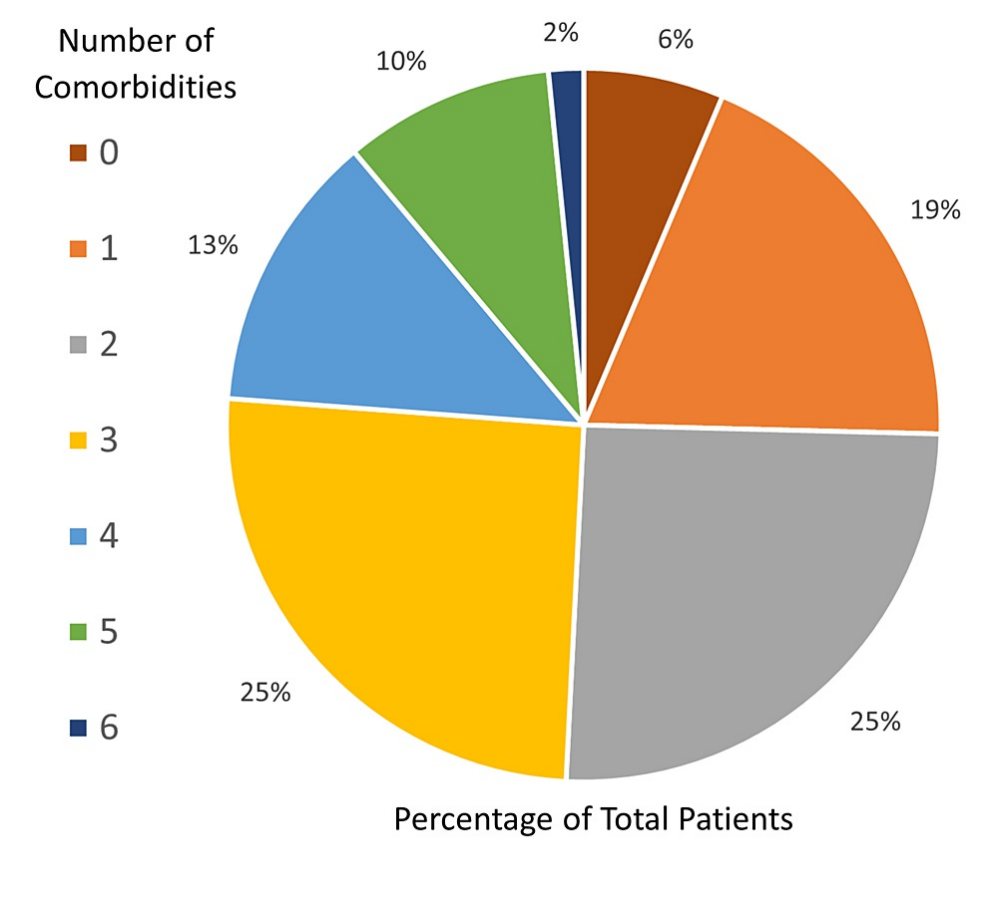
Percentage of comorbidities per patient. n = 63

**Figure 3:**
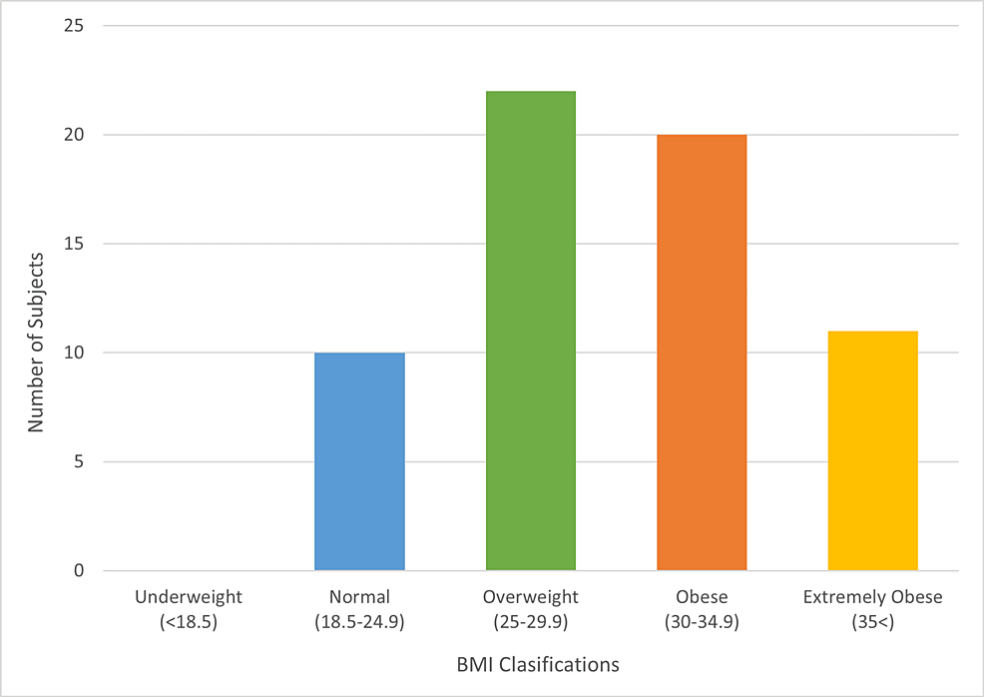
Subject body mass index reported according to the National Institute of Health classification. n = 63

Sixty-three of the 95 (66%) patient participants with one- to three-level interbody fusions were analyzed. The average age was 59 years old (range 24-82). Thirty-nine participants (62%) were female, and 24 participants (38%) were male. Surgeries included anterior lumbar interbody fusion (ALIF) 360º with posterior instrumentation in 48 cases (76%) and lateral lumbar interbody fusion (LLIF) 360º with posterior instrumentation in 15 cases (24%). Fifty-nine participants (94%) had at least one comorbidity, 47 (75%) had at least two, and 31 (49%) had at least three comorbidities (Figures 1-3).

The most common comorbidities included heart disease (43 participants, 68%), obesity (31 participants, 49%), and previous lumbar surgery (23 participants, 37%) (Table 1).

**Table 1:** Demographics and comorbidities ALIF: anterior lumbar interbody fusion, LLIF: lateral lumbar interbody fusion, SD: standard deviation

Characteristics	All participants		ALIF participants		LLIF participants	
	% or Mean ± SD	#/#	% or Mean ± SD	#/#	% or Mean ± SD	#/#
Age	59 ± 14.21	-	56 ± 13.69	-	72 ± 8.22	-
BMI	31 ± 6.03	-	32 ± 6.37	-	27 ± 3.04	-
Sex (female)	62%	39/63	56%	28/50	85%	11/13
Obesity	49%	31/63	44%	22/50	15%	2/13
Tobacco use	16%	10/63	58%	29/50	15%	2/13
Diabetes	17%	11/63	18%	9/50	8%	1/13
Heart disease	68%	43/63	10%	5/50	46%	6/13
Thyroid issues	19%	12/63	66%	33/50	77%	10/13
Respiratory issues	19%	12/63	20%	10/50	15%	2/13
Cancer	13%	8/63	30%	15/50	23%	3/13
Previous lumbar surgeries	37%	23/63	10%	5/50	38%	5/13

**Figure 4:**
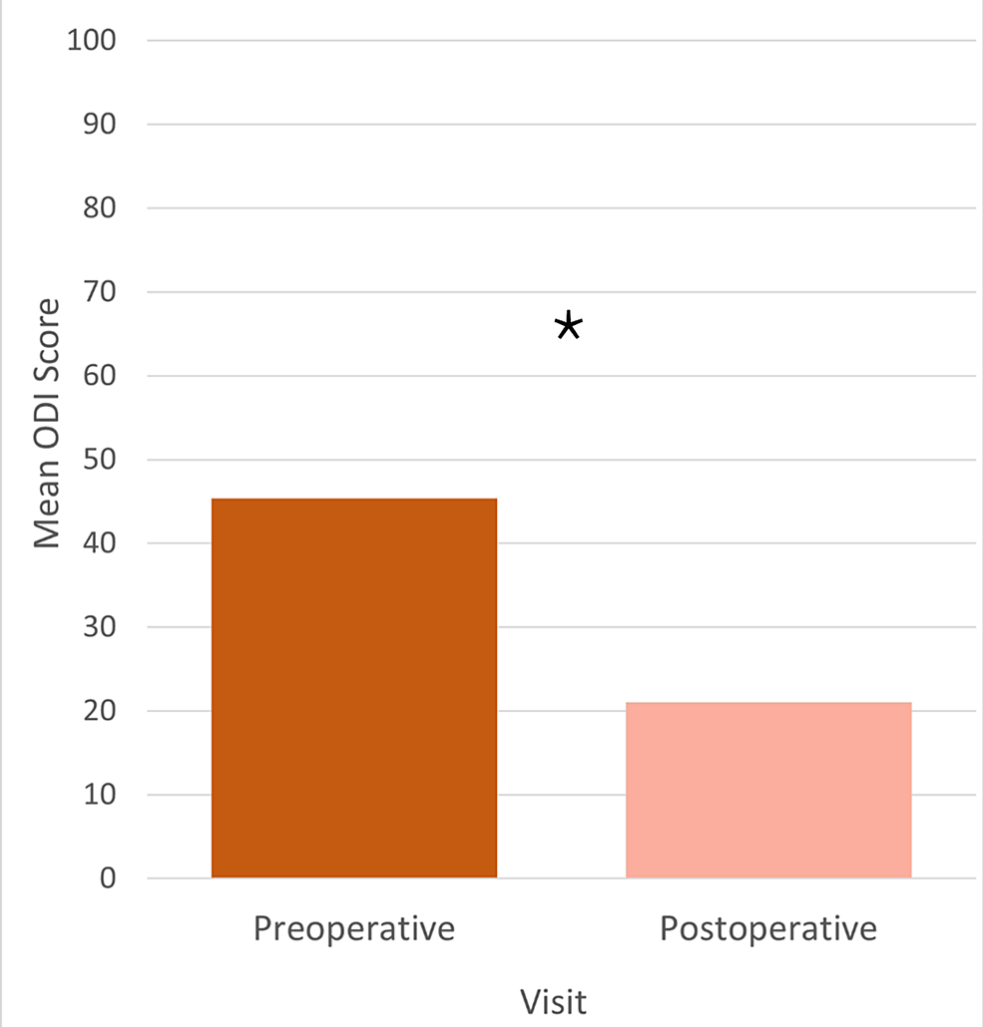
Oswestry Disability Index score grouped by preoperative and postoperative responses. * Meets the minimal clinically important difference (MCID) threshold; n = 63

The average length of stay (LOS) was two days (based on 45 participants). Primary indications for surgery included spondylosis (24 participants, 38%), spondylolisthesis (22 participants, 35%), lumbar scoliosis (nine participants, 14%), and spinal stenosis (eight participants, 13%). No study participants (0%) had reported complications, while two subjects (3%) had reoperation within one year of index surgery. Both secondary surgeries were indicated for adjacent-level symptoms: one for instability above the index procedure at 29 months postoperative and the second for stenosis below the index procedure at 13 months postoperative. A third patient declined a recommended intervention for junctional breakdown adjacent to the index level causing instability and radiculopathy. The percentage of participants with BMI considered obese (20, 32%) and extremely obese (11, 17%) was 49% (Figure 3). The mean preoperative ODI was 45, and the mean postoperative ODI was 21, with a mean ODI decrease of 24 (Figure 4). 

**Figure 5:**
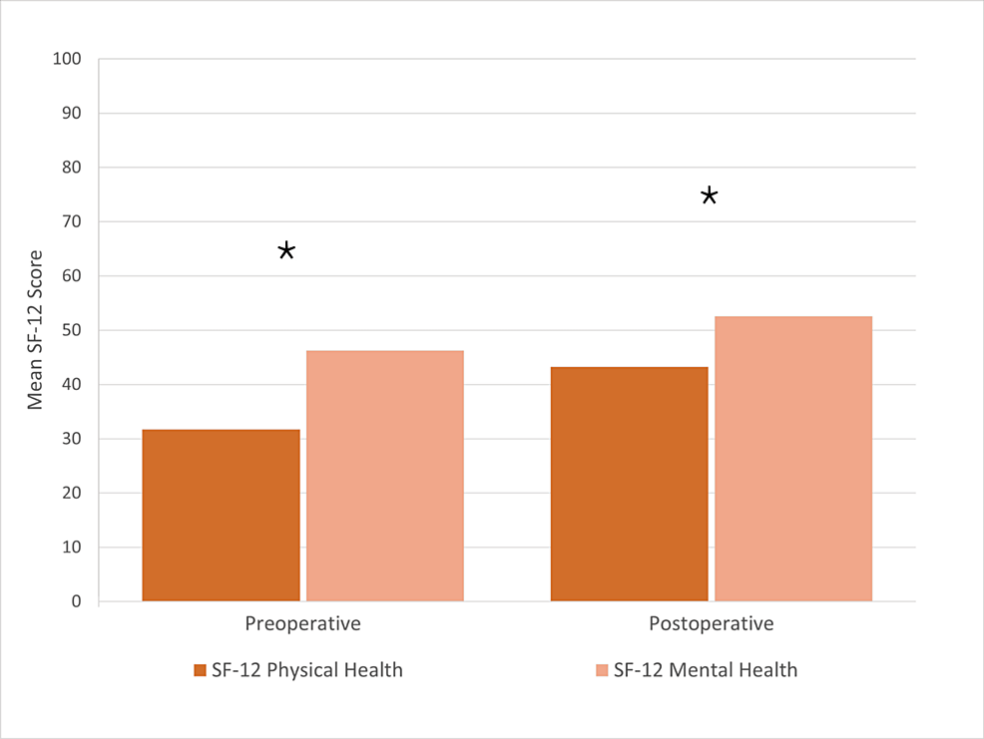
Short Form-12 Health Survey mental and physical health score at preoperative visit versus the mental and ohysical health score at postoperative visit. * Meets the minimal clinically important difference (MCID) threshold; n = 63

Both the SF-12 physical health scores and SF-12 mental health scores changed by 11.5 and 6.3, respectively (Figure 5).

**Figure 6:**
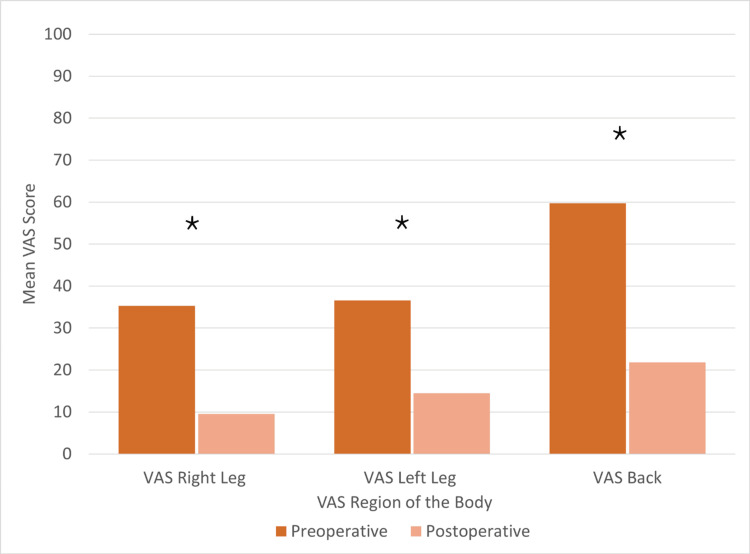
Visual analog scale score measured at the preoperative visit versus the postoperative visit. * Meets the minimal clinically important difference (MCID) threshold; n = 61

The mean VAS for the left leg, right leg, and back changed by a mean of 25.75, 22.07, and 37.87, respectively (Figure 6).

**Figure 7:**
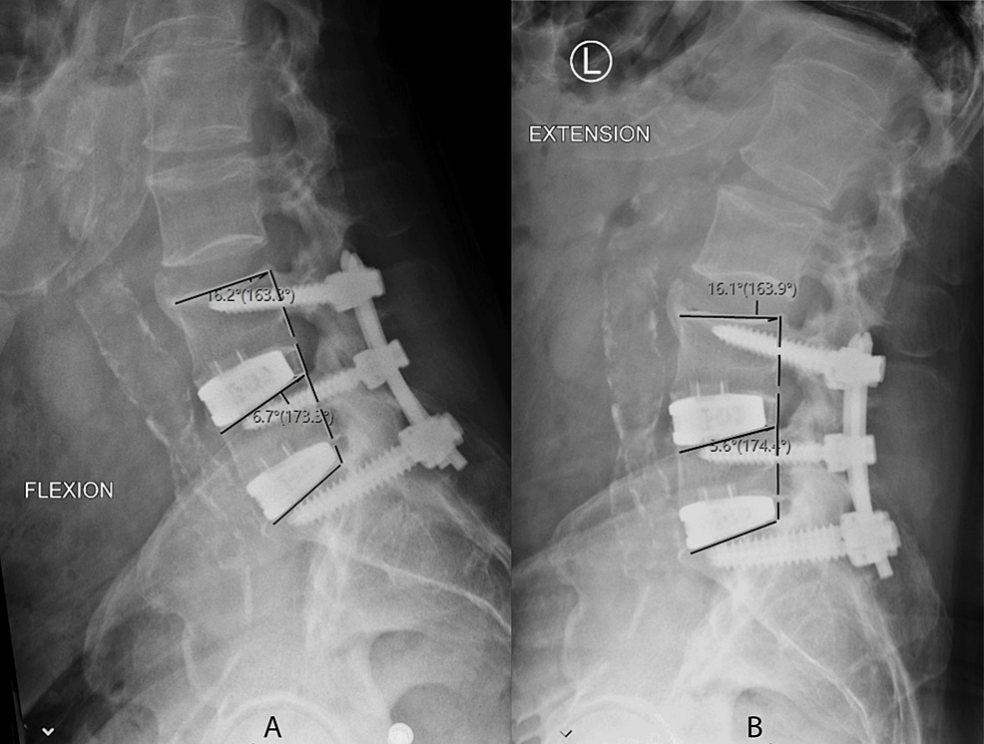
Flexion (A) and extension (B) radiographs demonstrating <3 degree of angular motion indicating a solid fusion of L4-L5, L5-S1 anterior lumbar interbody fusion.

**Figure 8:**
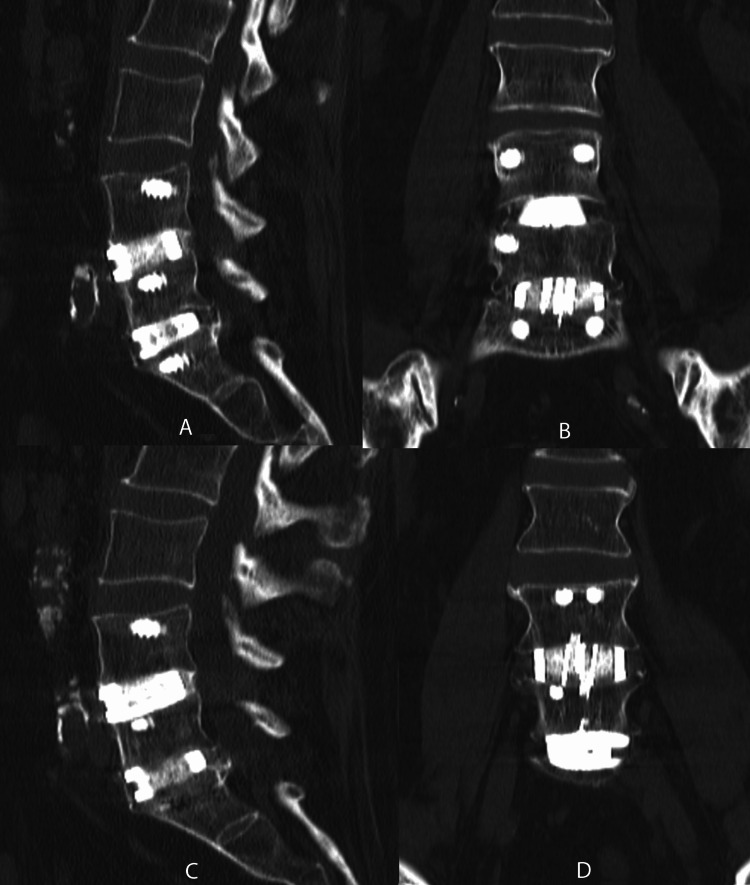
Sagittal (A, C) and coronal (B, D) CT imaging demonstrating complete fusion of L4-L5 and L5-S1 anterior lumbar interbody fusion utilizing BCP BCP: biphasic calcium phosphate

**Figure 9:**
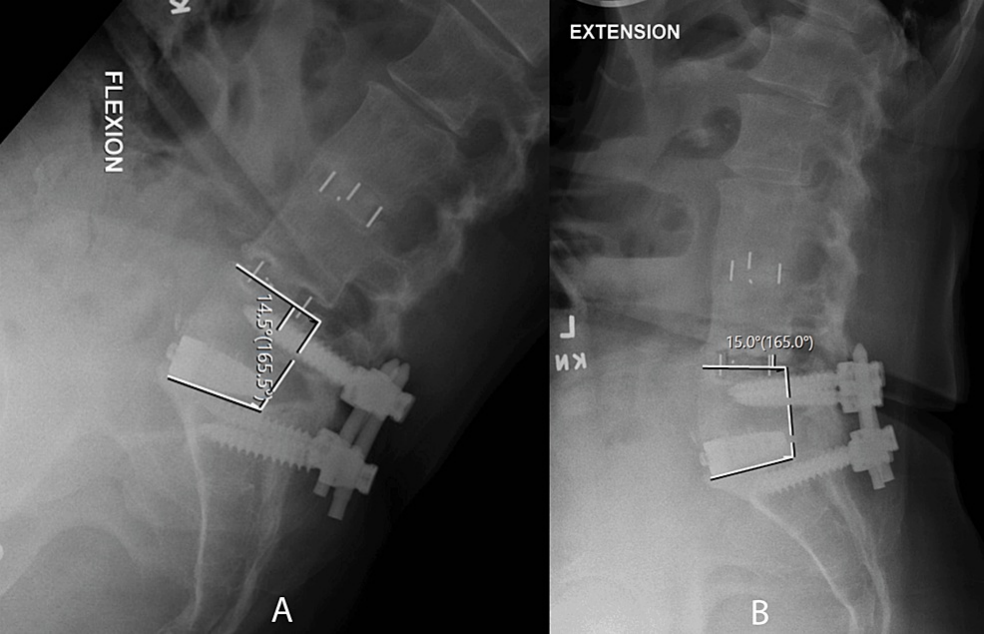
Flexion (A) and extension (B) radiographs demonstrating <3 degree of angular motion indicating a solid fusion.

**Figure 10:**
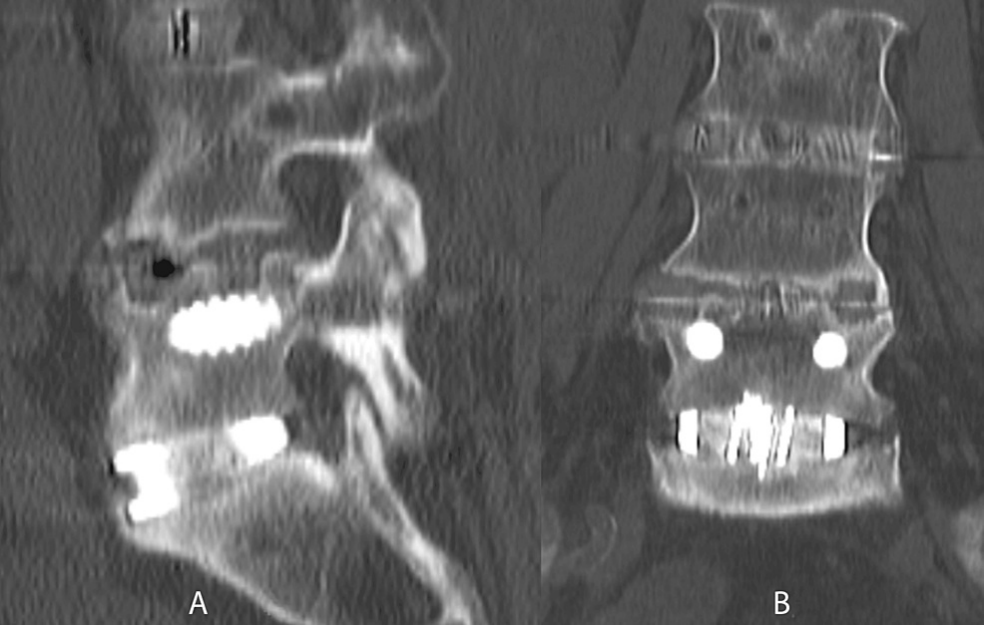
Sagittal (A) and coronal (B) CT imaging demonstrating a complete fusion and L5-S1 anterior lumbar interbody fusion utilizing BCP BCP: biphasic calcium phosphate

Radiographic outcomes included 63 patients and 101 levels who had CT scans at least one year (mean = 1.7 years) postoperatively. Of the 101 levels, 91 (90%) demonstrated complete bridging trabecular bone fusion with no evidence of supplemental fixation failure (see Figures 7-10 for the representative imaging).

Eight participants (13%) demonstrated cranial downgrowth and caudal upgrowth without bridging with no evidence of supplemental fixation failure, and one participant (1%) demonstrated complete bridging trabecular bone fusion with evidence of supplemental fixation failure. The per-patient fusion rate was 86%.

## Discussion

Bone graft substitutes have emerged as a crucial advancement in the field, serving as a viable alternative to autograft harvesting. They have played a pivotal role in improving patient outcomes by reducing operative times, minimizing patient morbidity, and achieving reliable fusions that rival autografts. While the majority of CaPs used in the United States are approved for posterolateral fusion, the growing body of evidence highlighting the benefits of advanced bone graft technologies has led to the recent clearance of CaPs for use in interbody fusion procedures.

Bone graft substitutes have continued to improve and advance over the past 30 years. In order to determine the efficacy of bone graft substitutes, high quality and quantity of scientific, pre-clinical, and clinical evidence must be obtained. In recent years, particular attention has been paid to surface topography and how it can interact with native cells to induce bone healing [20]. There has been extensive research into osteoimmunology, which has led to a new generation of bone graft substitutes. Most recently, CaPs have emerged as a viable alternative to autografts with comparable fusion rates. In particular, there have been numerous studies showing that BCP<µm can modulate mesenchymal stem cells and osteogenic precursor cells to differentiate into osteoblasts. BCP<µm affects the M1-M2 polarization of macrophages toward the M2 lineage and subsequently the pro-healing pathway to allow for bone ingrowth. The BCP<µm technology allows for new bone growth from the core and by creeping-edge repair, which can lead to a more reliable fusion. 

Utilizing BCP<µm, this study found a lumbar interbody fusion rate of 90% (91/101), based on fusion levels, and 86% (54/63) based on per patient. This is comparable to the gold standard of ICBG, with the advantage of decreased operative times, less patient morbidity from graft harvest, and decreased hospital LOS compared to ICBG harvest. This makes BCP<µm a viable alternative for lumbar interbody fusion.

A surgeon has multiple graft material options when performing lumbar interbody fusions, such as autograft, allograft, and synthetic materials. These include autologous iliac crest graft, autologous local morselized bone graft, bone morphogenic protein (BMP), decalcified bone matrix (DBM), and ceramic fillers. While each helps to achieve improved fusion rates, some materials present increased risk and/or morbidity. ICBG has been associated with higher rates of infection, donor site pain, hematoma development, and prolonged operative times [21]. BMP has been shown to produce significant inflammatory responses [22] and adverse structural changes, including ectopic bone formation. Although rare, the addition of DBM obtained from cadaverous tissue may result in disease transmission. Fusion constructs of a cage containing BCP ceramic used in a cervical application were found to have a lower rate of fusion than autogenous iliac bone graft and had a delayed fusion [23]. Calcium-based materials, such as calcium phosphate, tri-calcium phosphate, calcium sulfate, hydroxyapatite (HA), silica-substituted calcium phosphate, and bioglass having a high degree of porosity may restrict the mechanical strength and resorption rate [24]. In order to accurately assess pseudoarthrosis, a systematic evaluation utilizing both CT and radiographs reviewed by an independent surgeon must be utilized. Standardization of fusion assessment with CT scans at a minimum of one year post-operatively would help to decrease the ambiguity across the literature when it comes to the assessment of bone graft fusion status. In this study, fusion status was assessed with CT and radiographic evaluation by an independent spine surgeon. Fusion status was determined by bridging trabecular bone on CT with no evidence of hardware loosening or failure and less than three degrees of angular motion on flexion and extension radiographs. CT and flexion/extension radiographs have been demonstrated in a large systematic review to be the most reliable means of detecting pseudoarthrosis [25].

Pseudoarthrosis has been demonstrated in the literature to lead to poor patient satisfaction scores postoperatively [26]. In this study, patient-reported outcome measures had significant improvement from baseline levels to a minimum one-year postoperative. The mean ODI decreased to 24. Both the SF-12 physical health scores and SF-12 mental health scores improved by 11.5 and 6.3, respectively. The mean VAS scores for the left leg, right leg, and back improved by a mean of 25.75, 22.07, and 37.87 respectively.

Patient comorbidities have been shown to increase the risk of adverse events, decreased patient satisfaction scores, and reduced fusion rates. Fusion rates in patients with multiple medical comorbidities have been reported to be as low as 65% [27]. Depending on the condition and the severity of symptoms, adverse events may be managed conservatively or require additional surgical intervention. As an example, cauda equina syndrome, although rare, is severe and requires urgent surgical treatment [28], while other conditions, such as pseudoarthrosis, typically occur at a later time point from the initial surgery and are treated conservatively for a period of time, allowing for delayed healing. Pseudoarthrosis is a challenging complication in lumbar arthrodesis surgery and often occurs within one year of the index procedure [29]. It can lead to pain, instability, and additional surgeries. In this study, fifty-nine participants (94%) had at least one comorbidity, 47 (75%) had at least two, and 31 had at least three comorbidities. The most common comorbidities included heart disease (43 participants, 68%), obesity (31 participants, 49%), and previous lumbar surgery (23 participants, 37%). With this patient population, the study was able to achieve a 90% fusion rate per level and an 86% fusion rate per patient with no reported complications and improved VAS, ODI, and SF-12 scores. Patient optimization is essential to ensure more predictable fusion and improve patient outcomes following lumbar fusion surgery. In a properly optimized patient, bone graft substitutes, particularly BCP<µm, can play an essential role to help ensure successful fusion.

There are several limitations to the present investigation. The first limitation is that the study design utilizes a single-center consecutive cohort of patients rather than a randomized control with a possible introduction of bias in the selection of patients for participation. The authors attempted to mitigate this bias by inviting all patients who underwent the procedure to participate in the study (an “all comers” cohort) without strict eligibility criteria for patients to be included or excluded. Although ultimately there is the potential for patient self-selected bias, as with any clinical study, patients are free to accept or decline the invitation. Furthermore, although all potential patients were not available for follow-up, 66% of the patients invited opted to participate in the evaluation. The second limitation is the presence of a hardware artifact observed on CT during the assessment of fusion. This is a known limitation of utilizing CT in instrumented lumbar fusions. The adoption of titanium hardware and devices appears to lessen artifact degradation. To supplement the qualitative fusion assessment on CT, strict quantitative fusion as the criteria was implemented. The third limitation relates to the qualitative fusion and the margin of error. While dynamic fusion assessment on flexion and extension radiographs is widely accepted and relied upon in clinical trials as identified in the literature and within the FDA IDE for Spine Systems Guidance Document [30], there remains variability both in terms of intra- and interobserver assessments. Assessment of motion is also dependent on the quality of the radiographs; therefore, in an attempt to lessen this variable, all 12-month radiographic imaging was performed at one center under precise radiographic protocol. The fourth limitation is that the fusion environment may differ between surgical approaches. As previously discussed, studies have been conducted that compare outcomes for ALIF, LLIF, PLIF, and TLIF. While some publications found no statistical differences in clinical or radiographic outcomes between approaches, due to the anatomical and structural changes induced by different procedures, this procedure may not be applicable to TLIF or PLIF procedures. Lastly, due to the mixed cohort indications for surgery identified in the present study, the authors recognize this may be an additional confounder.

## Conclusions

Despite the limitations, the data presented in this real-world evidence study, evaluating the performance of BCP<µm in interbody fusions for degenerative lumbar spine disease, offer compelling evidence of successful fusion even within a challenging patient cohort. As more bone grafts gain clearance for interbody indication, surgeons and the industry must publish clinical data on the efficacy of bone grafts in this space. As far as we know, this is the first clinical data published on a bone graft of this category in interbody fusions. Ongoing studies are currently in progress to further enhance our understanding of this innovative graft substitute.
